# wholeskim: Utilising Genome Skims for Taxonomically Annotating Ancient DNA Metagenomes

**DOI:** 10.1111/1755-0998.70001

**Published:** 2025-07-21

**Authors:** Lucas Elliott, Frédéric Boyer, Teo Lemane, PhyloAlps and PhyloNorway consortia, Inger Greve Alsos, Eric Coissac

**Affiliations:** ^1^ UiT— The Arctic University of Norway Tromsø Norway; ^2^ The Arctic University Museum of Norway Tromsø Norway; ^3^ CNRS, Laboratoire d'ecologie Alpine (LECA) Université Grenoble Alpes Saint‐Martin‐d'Heres Rhône‐Alpes France; ^4^ CEA Genoscope National Sequencing Center Evry Paris Île‐de‐France France

**Keywords:** ancient DNA, environmental DNA, genome skims, k‐mers, metagenomics

## Abstract

Inferring community composition from shotgun sequencing of environmental DNA is highly dependent on the completeness of reference databases used to assign taxonomic information as well as the pipeline used. While the number of complete, fully assembled reference genomes is increasing rapidly, their taxonomic coverage is generally too sparse to use them to build complete reference databases that span all or most of the target taxa. Low‐coverage, whole genome sequencing, or skimming, provides a cost‐effective and scalable alternative source of genome‐wide information in the interim. Without enough coverage to assemble large contigs of nuclear DNA, much of the utility of a genome skim in the context of taxonomic annotation is found in its short read form. However, previous methods have not been able to fully leverage the data in this format. We demonstrate the utility of *wholeskim*, a pipeline for the indexing of k‐mers present in genome skims and subsequent querying of these indices with short DNA reads. *Wholeskim* expands on the functionality of *kmindex*, a software which utilises Bloom filters to efficiently index and query billions of k‐mers. Using a collection of thousands of plant genome skims, *wholeskim* is the only software that is able to index and query the skims in their unassembled form. It is able to correctly annotate 1.16× more simulated reads and 2.48× more true *seda*DNA reads in 0.32× of the time required by *Holi*, another metagenomic pipeline that uses genome skims in their assembled form as its reference database input. We also explore the effects of taxonomic and genomic completeness of the reference database on the accuracy and sensitivity of read assignment. Increasing the genomic coverage of the genome skims used as reference increases the number of correctly annotated reads, but with diminishing returns after ~1× depth of coverage. Increasing taxonomic coverage clearly reduces the number of false negative taxa in the dataset, but we also demonstrate that it does not greatly impact false positive annotations.

## 1 Introduction

Environmental DNA (eDNA) is a powerful tool for both palaeoecological reconstructions and contemporary biomonitoring (Laura Parducci et al. 2017; Taberlet et al. 2018). Targeted PCR amplification of conserved regions of DNA (metabarcoding) has been the most commonly used approach to identifying taxa in environmental samples (Von Eggers et al. 2022; Revéret et al. 2023). As the cost of DNA sequencing has decreased and the access to high‐performance computing clusters has increased, non‐targeted sequencing of the entire DNA content of a sample (metagenomic shotgun sequencing) has become a viable alternative (L. Parducci et al. 2019; Wang et al. 2021). This approach allows for the study of the complete taxonomic community present in a sample and the retrieval of genome‐wide loci (Graham et al. 2016; Slon et al. 2017; Zimmermann et al. 2023). However, currently both the available DNA reference libraries and bioinformatic tools may limit our ability to take full advantage of shotgun‐sequenced eDNA.

Compared to metabarcoding, taxonomic annotation is significantly more challenging with a shotgun approach, since the sequenced reads are not limited to a few loci. Instead, the reads are composed of sequences distributed throughout the entire genome, which necessitates the use of a reference database that ideally covers the whole genome of any potential organisms of interest. To date, the most comprehensive set of reference sequences usable for this metagenomic analysis is provided by the International Nucleotide Sequence Databases which is the collaborative project of GenBank, DNA Data Bank of Japan (DDBJ) and European Nucleotide Archive (ENA) (http://www.insdc.org). However, these are far from providing complete genomic sequences for all species. There are a number of ongoing initiatives to sequence and assemble the genomes of all known species on Earth (Gilbert et al. 2014; Lewin et al. 2018), but the timeline of these efforts extends decades into the future (Lewin et al. 2022). Until these projects are fully realised, low‐coverage, non‐targeted sequencing of all DNA extracted from an organism's tissues (genome skimming) can generate genome‐wide information for numerous taxa at low cost and effort (Straub et al. 2011; Coissac et al. 2016). The scalability of this approach is demonstrated by the PhyloNorway and PhyloAlps projects, which have sequenced representatives of the entire vascular flora of Norway/Polar Regions and the European Alps/Carpathians, respectively (Alsos et al. 2020).

In addition to the challenge of assembling a complete reference database composed of the whole genomes for all organisms of interest, metagenomic analysis also requires adapted algorithms to efficiently compare the large number of sequence reads produced for each sample to this terabyte‐sized database. The pipelines used in published studies to achieve taxonomic annotation of metagenomes rely on software belonging to two main categories: mapping software, such as *centrifuge* (Kim et al. 2016) or *bowtie2* (Langmead and Salzberg 2012), and diagnostic k‐mer based algorithms, such as *kraken2* (Wood et al. 2019). While these programs can index the complete genbank database, both approaches have difficulty achieving the same task on numerous genome skims. Mapping‐based software requires reference sequences to be significantly larger than the queried reads for efficient processing and to produce sensitive alignments, while current k‐mer approaches are unable to index the high k‐mer complexity of terabytes of short reads produced by genome skims.

Recent studies have demonstrated the feasibility of incorporating the genome skims produced by the PhyloNorway project into a reference database used to analyse shotgun‐sequenced ancient eDNA (Wang et al. 2021; Kjær et al. 2022). These studies indicated that the addition dramatically improved the proportion of metagenome reads that could be taxonomically annotated to genus‐level (Wang et al. 2021). These studies relied on the bowtie2, mapping‐based *holi* pipeline (Pedersen et al. 2016) as well as the assembly of PhyloNorway's genome skims using BBMap's tadpole (Bushnell 2014) and MEGAHIT (Li et al. 2015) to produce contigs with an average length of 496 bp (Wang et al. 2021). However, this preprocessing is computationally expensive and results in discarding information from low‐coverage regions of the genome. With PhyloNorway's genome skims averaging 0.5–1.0× depth of coverage (Alsos et al. 2020), the raw genome skims are losing a large amount of information during assembly which we hypothesize is useful for taxonomic annotation.

Here we present, *wholeskim*, a pipeline for annotating ancient DNA metagenomes consisting of very short reads by exploiting all the information contained in a set of unassembled genome skims. This is accomplished through two main steps: first, by building a reference database of these genome skims, each of which is associated with the set of k‐mers it contains; second, by annotating each read of the metagenome by comparing its k‐mer composition with the k‐mer reference database built in the previous step. Expanding on the functionality of *kmindex* (Lemane et al. 2023), wholeskim optimises storage and accuracy for a large number of input genome skims and performs least common ancestor assignment on each identified read. *Kmindex* leverages the Bloom filter data structure to efficiently index large metagenomic datasets and accurately queries them using the findere algorithm to significantly reduce false positive identifications (Robidou and Peterlongo 2021; Lemane et al. 2023).

In this paper, we demonstrate the functionality and benchmark the performance of *wholeskim* using 1541 genome skims from PhyloNorway and compare these measurements to the *holi* pipeline (Wang et al. 2021). We also examine the impact of reference database completeness on taxonomic assignment by considering two aspects: overall taxonomic coverage of the reference database and genomic completeness on a per species level. Finally, we demonstrate *wholeskim*'s ability to taxonomically annotate three sedimentary ancient DNA metagenomic datasets.

## 2 Methods

### 2.1 *wholeskim* Implementation

*wholeskim* consists of two bash scripts: *prep_indices*, which cleans the genome skims, groups them by k‐mer complexity, and finally indexes them, and *query_indices*, which queries the indices with a DNA metagenome and processes the resulting assignments. The input for *prep_indices* is a collection of genome skim data files in FASTA/FASTQ format. *query_indices* requires the previously built indices and a shotgun‐sequenced eDNA sample in FASTA/FASTQ format. The final output is a table with the following information for every read: the maximum proportion of shared k‐mers with a single database entry, the number of database entries considered for least common ancestor (LCA) assignment, and the taxonomic ID of the LCA. All code and docker image is available at https://github.com/ArcEcoGen/wholeskim.

### 2.2 Building of the k‐Mer Reference Database

Genome skims produced by the PhyloNorway project were used for reference database construction (ENA project number: PRJEB43865 (Alsos et al. 2020; Wang et al. 2021)). Prior to indexing, the 1541 genome skims were concatenated on a species level to produce 1323 separate entries for indexing. The programmes *centrifuge*, *bwa* and *kraken2* were all unable to index these 1.9 TB of unassembled genome skims due to requesting > 2 TB of memory during the course of construction. We were able to build the reference database using *kmindex* (Lemane et al. 2023), a software utilising Bloom filters to space‐efficiently index the k‐mers of large metagenomic datasets. A Bloom filter is a probabilistic data structure used to store numerous objects, here k‐mers, and to test whether an object is a member of that set. The reason for its effectiveness is that a Bloom filter is not based on an exact algorithm, but on heuristics. As a consequence of this characteristic, Bloom filters can produce false positives, answering that the k‐mer is a member of the set when it is not, but they cannot produce false negatives. To reduce the number of false positives, *kmindex* relies on the corrective *findere* algorithm (Robidou and Peterlongo 2021; Lemane et al. 2023) which queries *z* neighbouring k‐mers of size k—z + 1, instead of a k‐mer of size k. It only reports a positive match if all *z* k‐mers are indexed in the reference database. Thus, if the Bloom filter has a false positive rate *fp*, the resulting false positive rate after using the *findere* algorithm is *fp* ^*z*^.

*wholeskim* builds the k‐mer reference database in a two‐steps process. First, it filters out from the genome skims all the sequence reads that match to commonly suspected DNA contaminants in a genome skim (bacteria, fungi and human DNA) and are not found in a representative set of other related reference genomes. Second, it builds a Bloom filter for each genome skims with the cleaned reads.

#### 2.2.1 Genome Skim Cleaning

Prior to indexing, *wholeskim* filters out from the genome skims all reads that match to a commonly suspected DNA contaminant in a plant tissue extract. The following large taxonomic groups are indexed using *kmindex*: Bacteria (NCBI taxonomy ID: 2), fungi (4751), algae (2763), and *Homo sapiens* (9606). A last bloom filter corresponding to our group of interest Viridiplantae (33090) is also built. Using the taxonomic annotation procedure described below, only reads unidentified or annotated as Viridiplantae are conserved. Here, these cleaning bloom filters are built from genbank's 259 release.

#### 2.2.2 k‐mer Reference Database Indexing

Bloom filters encode k‐mers as a bit field (an ordered set of 0s or 1s). The false positive rate of a Bloom filter depends on the number of bits (m) used to encode the k‐mers and the number of different k‐mers stored in the filter. To obtain a given false positive (fp) rate when storing n k‐mers, m is defined according to Formula (1).

(1) $$m=\frac{-n}{\ln\left(1-\mathrm{fp}\right)}$$

We aim for a *fp* = 0.05 for each bloom filter, corresponding to an overall false positive rate at query‐time of 0.05^3^ = 1.25 × 10^−4^. *wholeskim* works with k‐mer of size k = 34 as a tradeoff between specificity, efficiency and the ability to annotate the majority of reads present in ancient DNA metagenomes (Figure S1). To optimise its computation and storage footprint, kmindex allows for simultaneous indexing of multiple bloom filters with the same bit size (m). To minimise storage and maintain a consistent *fp* rate, wholeskim groups genome skims for indexing based on their k‐mer diversity, the count of distinct k‐mers present in each skim estimated by *ntcard* (Mohamadi et al. 2017).

### 2.3 Assigning a Taxon to a Read From a Metagenome

The decision to assign a taxon to a query read, Q, is based on the number of shared k‐mers, S_G_, it has with each of the genome skims, G. The assignment algorithm is:

- Calculate N_Q_ = L_Q_—k + 1, the total number of k‐mers present in Q, a read of length L_Q_.
- Identify S_max_, the maximum number of k‐mers shared between Q and any of the indexed genome skims G.
- Calculate t_max_ = S_max_/N_Q_.
- If tmax ≥ tc, the cutoff proportion for a positive match, define t_min_ = t_max_–Δ, a threshold for similar matches.
- Calculate S_min_ = t_min_ × N_Q_.
- Select all genome skims G with S_G_ ∈ [S_min_; S_max_].
- Assign to Q the lowest common ancestor (LCA) of all taxa associated with the selected genome skims, using the NCBI taxonomy as a reference.

To reduce the noise of assignments, only assignments to taxa that appeared in greater than a proportion, r, of the total reads were retained. After optimization through testing with simulated datasets, the three parameters of the procedure have been set to t_c_ = 0.7, Δ = 0.1, *r* = 10^−5^ (Figure S2). The optimal t_c_ and Δ parameters were determined by querying simulated reads at various cutoff proportions and maximising the ratio of correct species level assignments to off‐target assignments (see Sensitivity and specificity tests, Figure S2). The r parameter was set at 10^−5^ to the discard the expected false positive assignments from the bloom filter calculated to be 1.25 × 10^−4^.

### 2.4 Evaluating Wholeskim Performances

**FIGURE 1:**
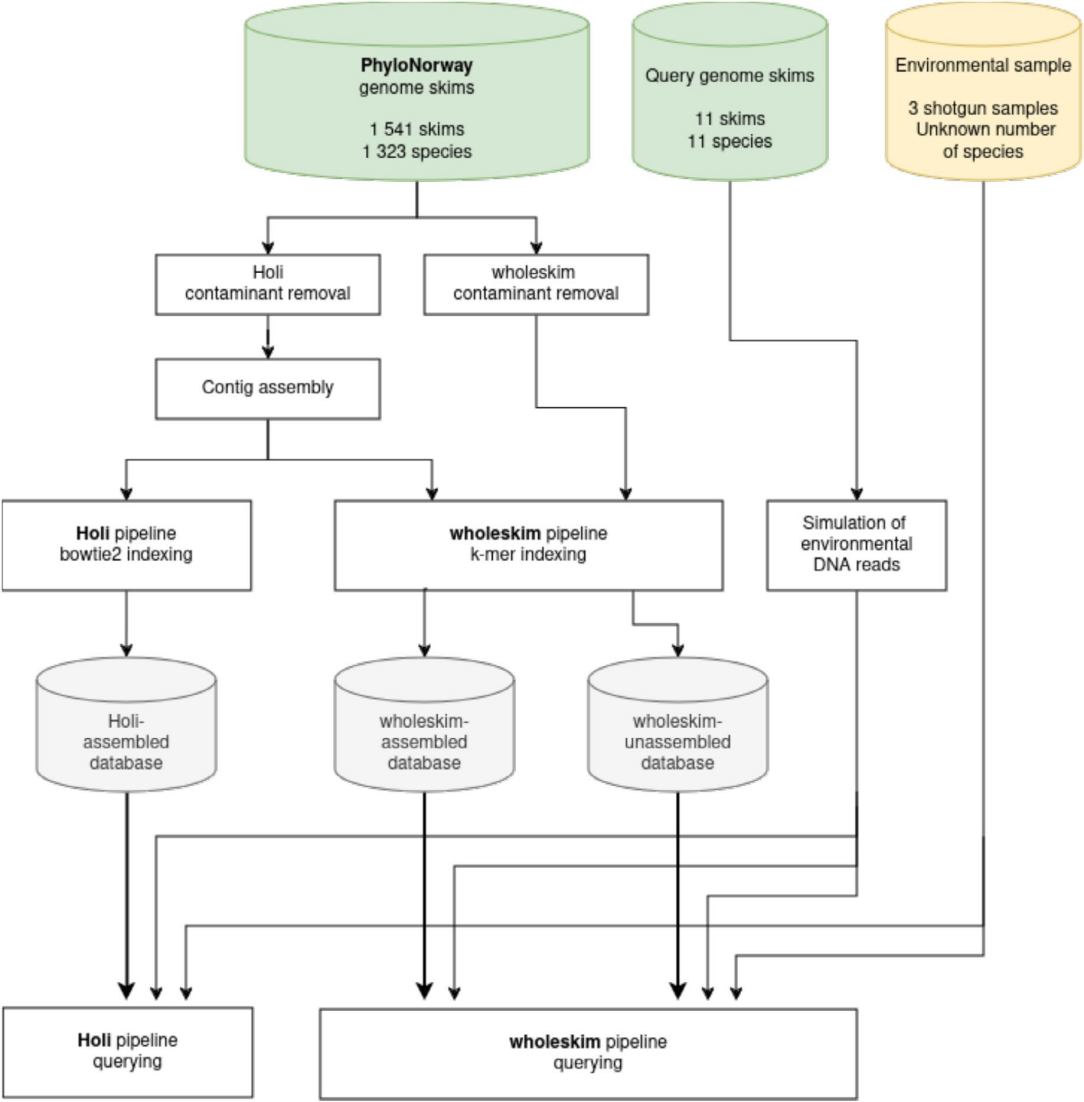
Diagram of the three different workflows that were evaluated. From left to right: The Holi pipeline tested with the assembled contigs, wholeskim tested with the assembled contigs, and finally wholeskim tested with the unassembled reads.

Wholeskim was evaluated on two aspects (Figure 1). (1) Its efficiency in indexing and querying the subset of 1541 PhyloNorway genome skims released in (Wang et al. 2021). This efficiency was measured in terms of indexing speed, querying speed, and memory requirements. (2) The sensitivity and specificity of wholeskim in annotating sequences were compared with those of the Holi pipeline (Wang et al. 2021; Kjær et al. 2022). These estimates of the sensitivity and specificity of wholeskim were made considering the impact on the completeness of the reference database in terms of taxonomic coverage and sequencing depth for a given species. Computations were performed on an HPC cluster node with 2 × 16‐core Intel Xeon Gold 6130 processor 2.10 GHz with 192 GB of memory. Databases were stored on an SSD to facilitate faster read access since *kmindex* does not load the full database into memory.

#### 2.4.1 Sensitivity and Specificity Tests

Two of the skims used for querying the pipeline, *Thesium alpinum* (PHA009155) and *Salix retusa* (PHA007876), are taken from PhyloAlps while the other nine ( *Avenella flexuosa* , *Betula nana* , *Betula pubescens* , *Bistorta vivipara* , *Caltha palustris* , *Dryas octopetala* , *Picea abies* , *Pinus sylvestris* , *Vaccinium uliginosum* ) were extracted/sequenced in Tromsø following the PhyloNorway protocol (Alsos et al. 2020). None of these skims were included in the assembled or unassembled reference databases. Sets of 200,000 reads were simulated from each of these 11 genome skims using the *adrsm* software (Borry 2018). Read simulation consisted of reducing the actual length of the sequencing reads of the genome skims to mimic the size distribution observed in ancient DNA metagenomes (mean insert size set to 35 based on median age samples from (Pedersen et al. 2016)). The simulated reads from each species were run through the pipeline separately.

#### 2.4.2 Comparison of Sensitivity and Specificity between the *Holi* and *wholeskim* Pipelines

The annotations from both pipelines were sorted into eight categories. Four categories describe different levels of precision in an accurate assignment: (i) “target species” when the read is annotated as the correct species, (ii) “target genus” when the read is annotated at the genus‐level only, but the correct one, (iii) “target family” when the read is annotated at the family‐level only, but the correct one and (iv) “higher target level” when the read is annotated at a taxonomic level higher than family, but the correct one. Four categories correspond to different levels of misclassification: (i) “incorrect species/target genus”, when the read is annotated to a different species in the target genus, (ii) “incorrect genus/target family” when the read is annotated to a different species or genus in the target family, (iii) “incorrect family”, when the read is annotated to a *Viridiplantae* clade outside the previous two categories and (iv) “unidentified” when a read is not assigned.

Three workflows were used to taxonomically assign reads: (i) the *Holi* pipeline using the assembled contigs from 1541 PhyloNorway genome skims downloaded from Dataverse.no (doi.org/10.18710/3CVQAG), (ii) the *wholeskim* pipeline using the unassembled 1541 genome skims and (iii) the *wholeskim* pipeline using the assembled contigs from workflow (i).

Workflow (i) was executed by following the scripts present in https://github.com/miwipe/KapCopenhagen (Kjær et al. 2022) and using only the 1541 PhyloNorway assembled genome skims as a reference database. An overview of this workflow is presented in Figure S3. To harmonise comparison with the *wholeskim* pipeline, reads assigned by *Holi* to taxa comprising less than a cutoff proportion (*r* = 10^−5^) of the entire dataset were set to unidentified, a generally more conservative threshold than the cutoff proportion of (*r* = 10^−5^) for only annotated reads as recommended by (Pedersen et al. 2016).

We break down the assignments of four species in detail that represent varying levels of representation in the reference database. They were selected as follows: *Betula nana* was selected as a well‐represented taxon because six skims of the *Betula* genus, including four from *Betula nana* , are present in the reference database; *Avenella flexuosa* was selected because, it is the only member of the *Avenella* genus present in the reference database; *Salix retusa* was chosen as the species is not represented in the reference database, but 39 other *Salix* skims representing 30 species are present; and finally for *Thesium alpinum*, no members of the Santalaceae family were present in the reference database. Workflows (i) and (ii) are further contrasted by summing the eight categories of taxonomic assignments across all nine test species that are represented in PhyloNorway and constructing a confusion matrix. This allows us to assess the overall performance of the two pipelines and investigate how reads classified by one workflow are assigned by the other.

#### 2.4.3 Impact of the Genome Coverage on Assignment Quality

Seven genome skims of *V. uliginosum* totalling 68 M reads are included in the 1541 skims of PhyloNorway. With an estimated genome size of 600 MB (Sultana et al. 2020), this represents an expected maximum genome depth of coverage of 11×. To assess the impact of genome coverage and database completeness on *whole skim*'s accuracy, the simulated *V. uliginosum* eDNA reads were assigned using the following subsets of the reference database: a database excluding all Ericaceae genome skims, a database excluding all *Vaccinium* genome skims, and databases including 0, 1, 2, 3, …, 19, 20, 24, 28, …, 68 M reads of *V. uliginosum* .

### 2.5 Comparative Efficiency of Wholeskim and Holi on True Ancient Metagenomes

Three shotgun‐sequenced *sed*aDNA samples from northern Norwegian archaeological midden complexes were annotated with both the *Holi*‐assembled and *wholeskim*‐unassembled workflows. The samples (GB‐6, GB‐5 and IG‐4) were dated to 4.2, 4.0 and 0.7 ka respectively and extracted using the DNeasy PowerLyzer PowerSoil kit (Qiagen: 12855‐100; Komatsu et al. in prep). Libraries were prepared for paired‐end sequencing using single‐stranded library preparation designed specifically for highly degraded ancient DNA (Komatsu et al. in prep). The paired‐end sequenced reads were merged, adapter sequences were removed, and reads less than 34 bp were discarded using *fastp* v0.23.4 (Chen et al. 2018). The resulting 13.9, 16.7 and 17.5 M reads respectively were annotated using the *Holi‐*assembled and *wholeskim*‐unassembled workflows. Taxa with less reads than 0.001% of the total were discarded from both workflow's final assignments. Assignments at species level were collapsed to genus‐level as this is a more reliable level of identification for shotgun data (see results), while assignments to taxa above family‐level were discarded. Additionally, a two million years old permafrost *seda*DNA sample from Greenland was processed through the *wholeskim* pipleine (sample 119_B3_116_L0, ERR10493283, Kjær et al. 2022). Reads taxonomically annotated to the *Betula* genus from this sample were mapped to an assembled *Betula pendula* genome (GenBank ID: GCA_900184695.1) through *bwa mem* (Li 2013) and the resulting damage pattern was quantified using mapDamage v2.2.1 (Jónsson et al. 2013).

## 3 Results

### 3.1 Genome Skim Cleaning

**FIGURE 2:**
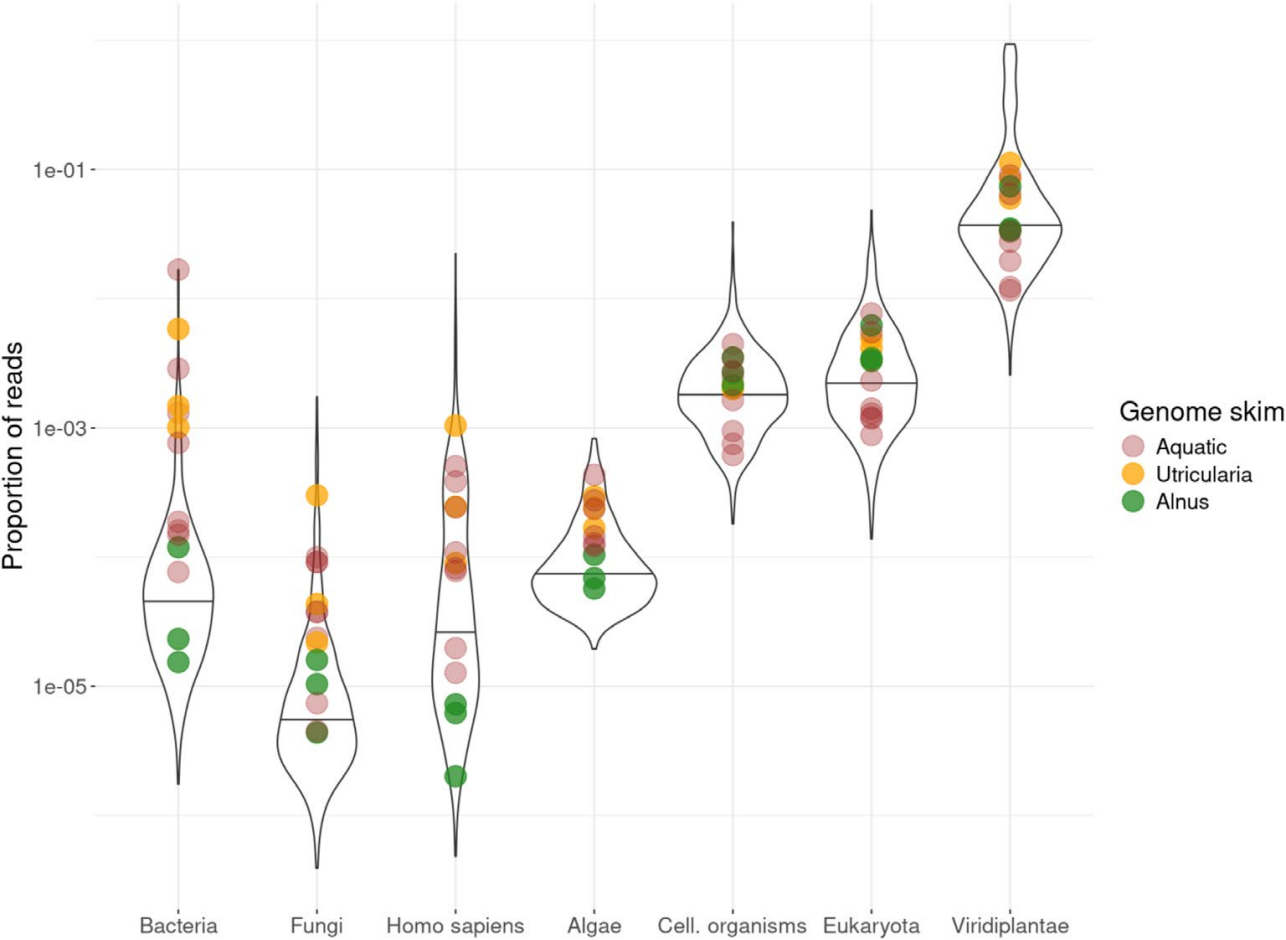
Violin plot of the proportion of identified reads for each PhyloNorway genome skim used to construct the reference database. Reads identified to the groups Algae, Bacteria, Fungi, or *Homo sapiens* were discarded as putative contamination. The genome skims of a subset of aquatic plants and *Utricularia* are highlighted as possessing more than average amounts of contaminated reads in comparison with a common terrestrial taxon *Alnus*.

The genome skims produced by PhyloNorway had a mean value of 4.64 million read pairs (sd = 1.58 million, Alsos et al. 2020). The cleaning step of wholeskim reduced the size of these raw genome skims by a median value of 0.015%. Among the rejected reads, median percentages of 0.007%, 0.004%, 0.002% and 0.0005% were identified as algae, bacteria, fungi and human contamination (Figure 2). However, a few genome skims of mostly aquatic plant taxa had considerably more contamination detected with maximum values for the previous categories of 0.08%, 1.7%, 2.2% and 0.18%. By comparison, terrestrial taxa such as *Alnus* had values more in line with the median proportions of contamination (Figure 2). Among the selected reads, a median value of 3.7% was identified by the filter as Viridiplantae reads, while the remaining ~96% matched none of the tested categories and were considered as not yet sequenced part of the plant genome.

### 3.2 Genome Skim Information Content

**FIGURE 3:**
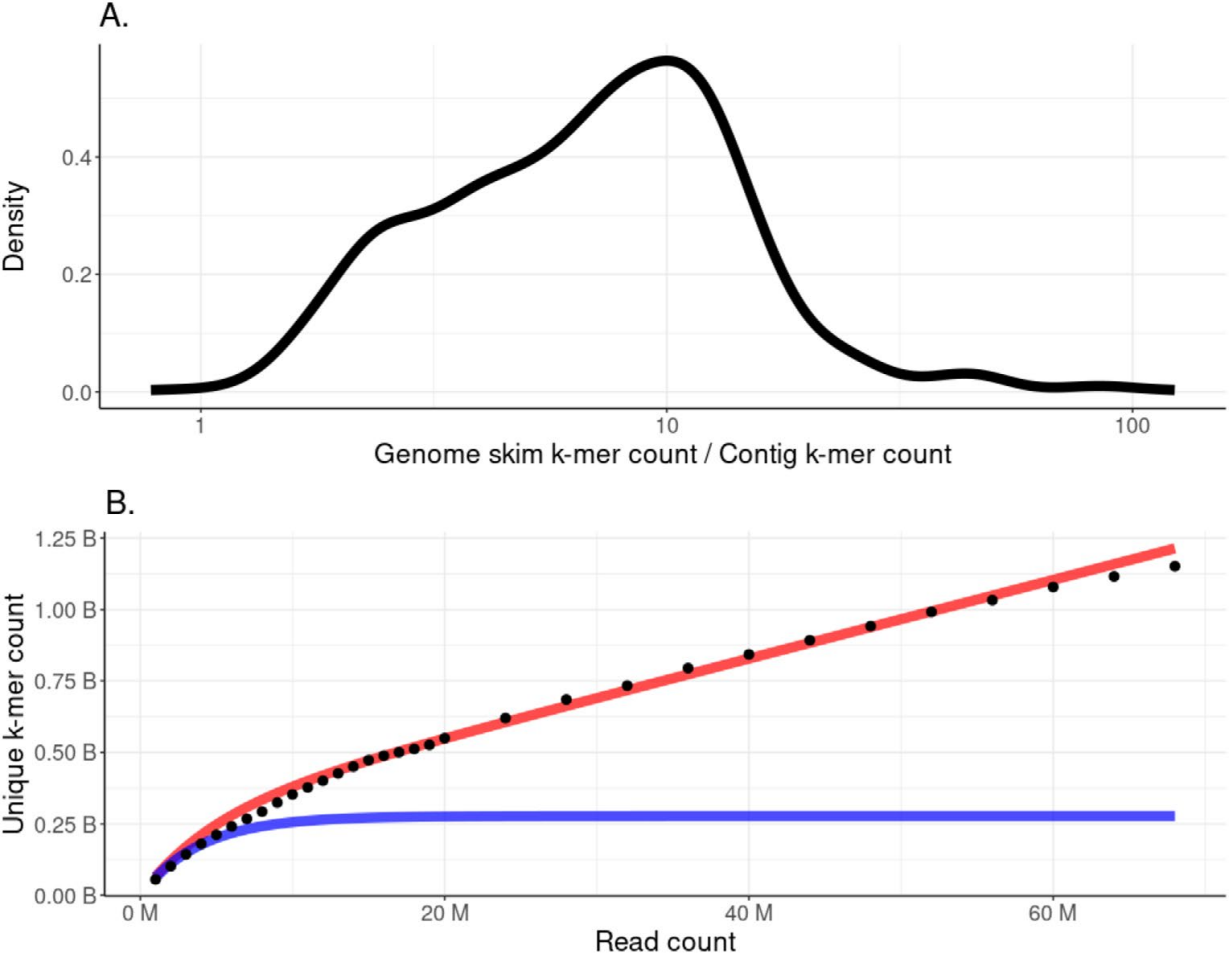
(A) Distribution of the ratio between the number of distinct k‐mer (*k* = 31) associated with a species in the genome skims dataset and the equivalent number in the corresponding contig dataset. (B) The points represent the number of unique k‐mers (*k* = 31) for a given number of reads for *Vaccinium uliginosum* from the PhyloNorway genome skims. The blue line represents the expected number of unique k‐mers found in an unassembled genome skim for a genome of *V. uliginosum* 's size (600 MB) and read size of 101 bp. The red line shows the same relationship, but with an introduced sequencing error rate of 4.4 × 10^−3^.

The information content of skims and contigs was measured by grouping unassembled genome skims or contigs by species and then counting the number of distinct k‐mers (k = 31) present in each set. The ratio of the number of k‐mers in skims to the number of k‐mers in the corresponding contigs for a species varies from 1 to 100 with a mode around 10 (Figure 3A). While a portion of the distinct k‐mers present in the unassembled genome skims are due to sequencing error, there is still a drastic reduction in information content caused by the skim assembly step required by mapping‐based approaches such as the *Holi* pipeline. Unassembled genome skims with a higher depth of coverage produce increasing numbers of distinct k‐mers as expected, but this number does not begin to plateau after reaching > 1× coverage (Figure 3B). The observed number of unique k‐mers found in the *V. uliginosum* skims matches the expected number of unique k‐mers from given a sequencing error rate of 4.4 × 10^−3^ (Figure 3B, equation given in Supplementary).

### 3.3 Performance of *Wholeskim* Compared to *Holi*

#### 3.3.1 Indexing Efficiency

The indexing of the 1541 genome skims or 1.9 TB of sequences by *wholeskim* required 11.8 h of computation time with a maximum of 54.0 GB of random access memory (RAM) used. To this, 7.6 h per billion reads of the reference genome skims for the cleaning step have to be added. The *Holi* pipeline required 36.4 h of computation time to index (through bowtie2‐build) the 152 GB of contigs requesting a maximum of 240.0 GB of RAM. An unknown amount of time was used to assemble and preprocess the contigs used as input. The resulting index created by *wholeskim* required 472 GB of storage, larger than the 311 GB occupied by *Holi*'s index.

#### 3.3.2 Querying Efficiency

A total of 2,187,986 simulated environmental DNA sequences from eleven species were taxonomically annotated using both the *wholeskim* and *Holi* pipelines. The querying of these reads by *wholeskim* with the unassembled genome skims required 3.1 h and a maximum of 4.8 GB of RAM. By comparison, *Holi* required 3.7 h to index the same set of reads while loading significant portions of the reference database in memory and requesting a maximum of 115 GB of RAM.

### 3.4 Overall Sensitivity and Specificity of Workflows

**FIGURE 4:**
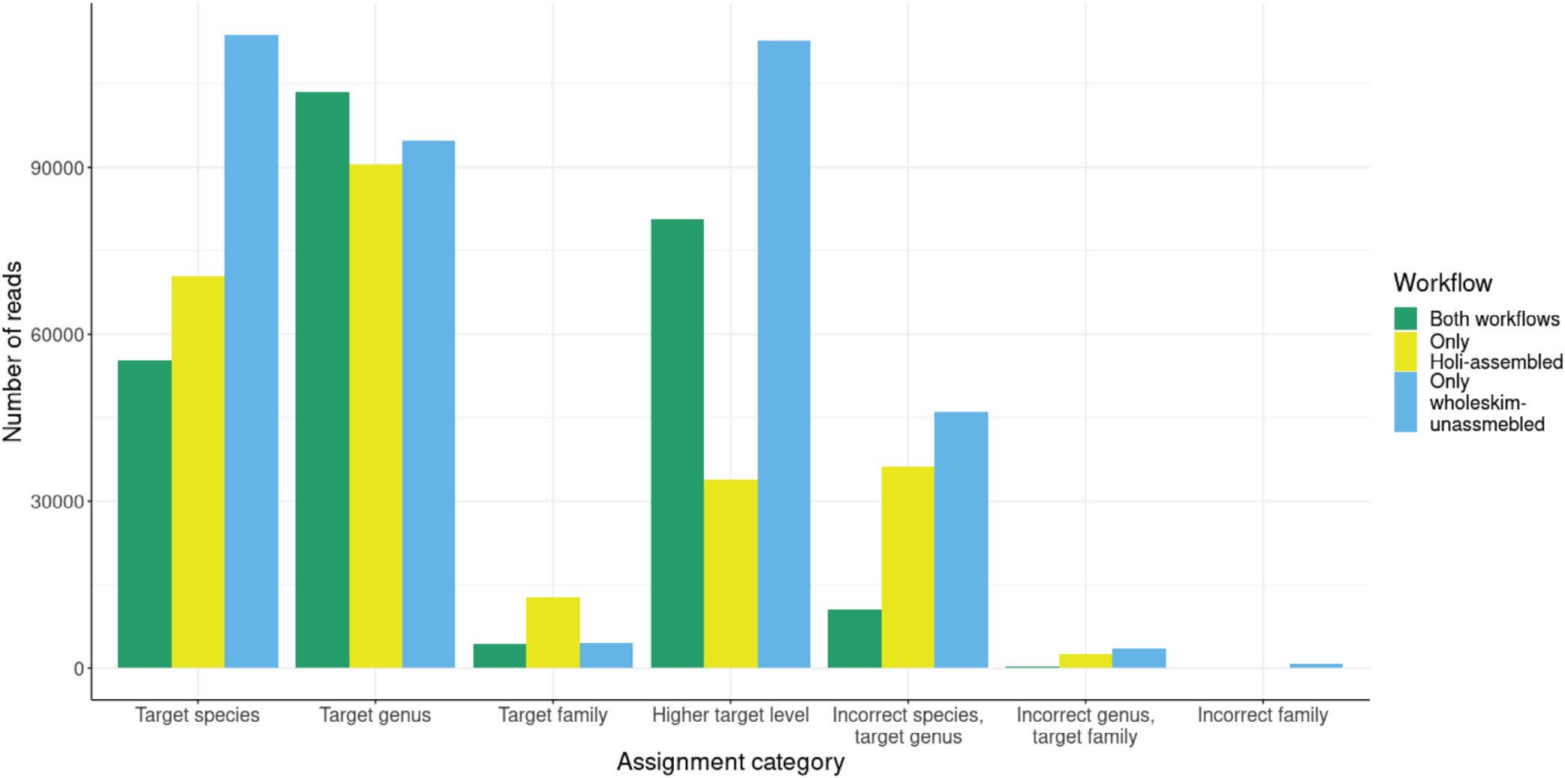
The overlap of simulated reads identified by the *Holi*‐assembled and *wholeskim‐*unassembled workflows. The set of reads is composed of all nine test species present in the PhyloNorway reference database.

The *wholeskim*‐assembled workflow performed the worst, misassigning more reads and correctly identifying less reads for every taxon than both other workflows (Figure S4). Due to this poor performance, we exclude this workflow from future comparisons. The *wholeskim*‐unassembled and *Holi*‐assembled workflows both correctly annotated a plurality of reads for each species with few misassignments. Averaged over the simulated reads from the nine species that are present in PhyloNorway, *wholeskim*‐unassembled correctly identified 20.2% of the reads at a species or genus‐level while *Holi*‐assembled correctly identified 15.3% of the reads at these levels. At the species level, *wholeskim*‐unassembled assigns nearly 1.34× more reads than *Holi*‐assembled. At the genus‐level, *wholeskim*‐unassembled assigns 1.16× more reads. This difference is even larger for correct assignments at the taxonomic level of family or above, but these identifications are rarely useful for metagenomic annotation purposes. *Holi‐*assembled and *wholeskim‐*unassembled incorrectly assign 3.0% and 3.4% of reads respectively with > 90% of these misassignments to a congeneric species of the target. There is little overlap in the sets of reads that each workflow annotates with only 21.6% of the total reads annotated to target species shared between workflows (Figure 4).

### 3.5 Taxonomic Completeness of Reference Database

**FIGURE 5:**
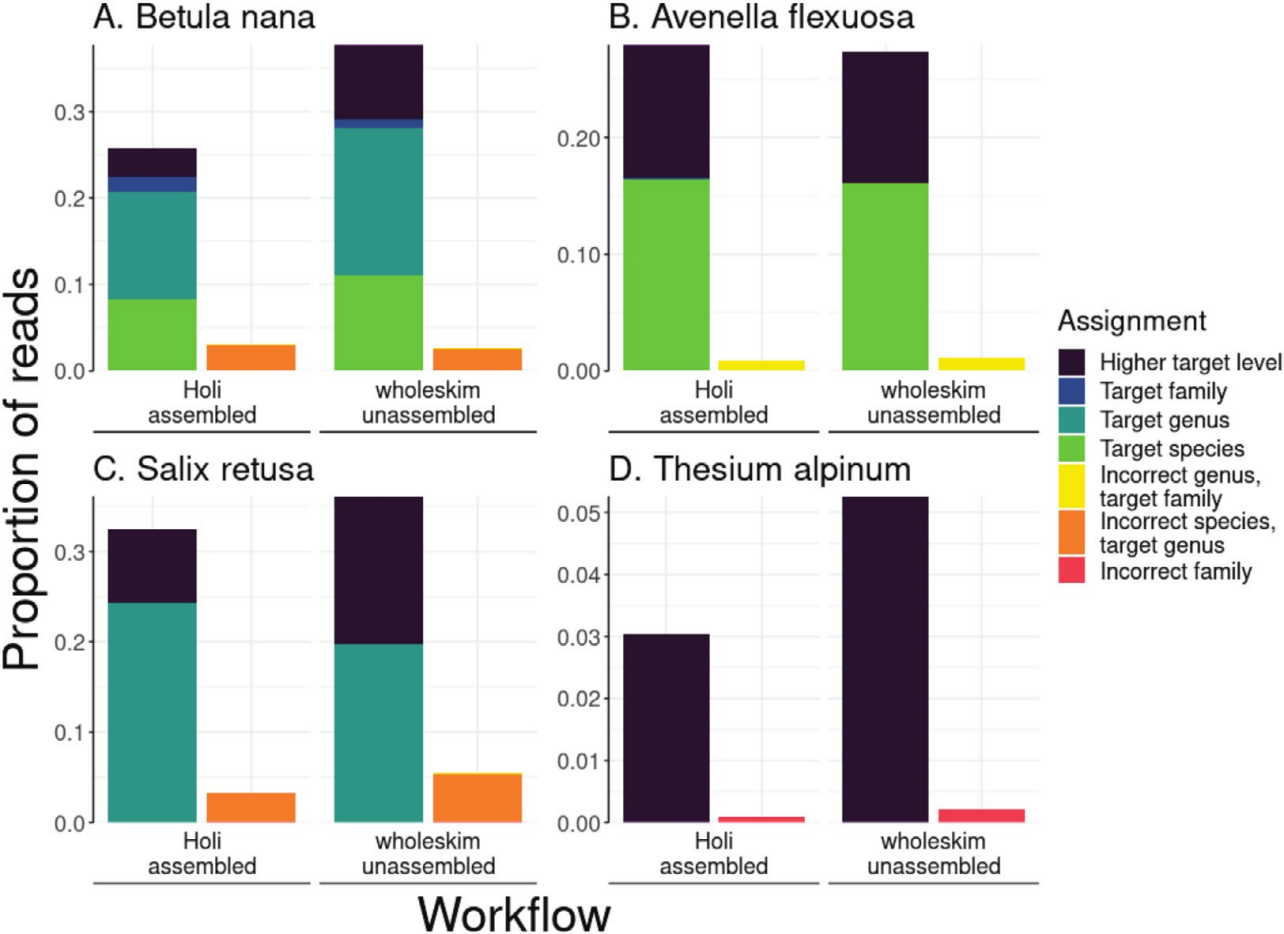
The effect of taxonomic completeness of the reference database on assignment for each workflow. Each workflow's assignments are divided into two stacked bars representing correct and incorrect assignments which are further broken down by colour according to the legend. The represented species are (A) *Betula nana* which has 4 genome skims in the database along with 2 more skims from the *Betula* genus, (B) *Avenella flexuosa* which is present with one species in the database, and it is the only species within this genus in the database, (C) *Salix retusa* which is not present in the reference database, but skims of 38 other *Salix* species are included and (D) *Thesium alpinum* which is not present in the database, and no members of the Santalaceae family are in the reference database. Note the differences in scale of the y‐axis.

Both the *Holi‐*assembled and *wholeskim*‐unassembled workflows accurately annotated reads from species with varying levels of representation in the reference database. *Betula nana* , a well‐covered species and genus, had 28.1% and 20.6% of reads assigned correctly to genus or species level by *wholeskim‐*unassembled and *Holi*‐assembled (Figure 5A). Only 2.4% and 3.0% of reads were incorrectly assigned by each workflow respectively, and all of them were to another species in the *Betula* genus. *Avenella flexuosa* is the only member of the *Avenella* genus present in the database and consequently the only misassignment is 1.1% and 0.8% of reads to taxa in the same family (Figure 5B). Both workflows correctly assigned 16.1% of this species' simulated reads to the species level. *Salix retusa* had no skims present in the reference dataset, so no reads were assigned correctly to species level, however 24.2% and 19.7% were assigned to the *Salix* genus. The workflows assigned 3.2% and 5.2% to other *Salix* species and < 0.1% to taxa outside the Salicaceae family. Without any representatives of the family Santalaceae in the reference database, 95% of *Thesium alpinum* reads were left unidentified while < 0.2% were incorrectly annotated by both workflows.

### 3.6 Genomic Completeness of Reference Database

The effect of genome skim sequencing effort on taxonomic annotation accuracy was tested with the *wholeskim*‐unassembled workflow by constructing the reference database using increasing numbers of *V. uliginosum* reads. As the number of genome skim reads, and consequently the number of unique k‐mers, increased in the reference database, the proportion of correctly annotated simulated *V. uliginosum* reads also increased (Figure 5). This trend is linear until 600 million unique k‐mers, roughly corresponding to genome size of *V. uliginosum* (Sultana et al. 2020), where it continues to increase, but with a smaller slope. The proportion of unidentified and misassigned reads follows a similar, but inverted trend as the number of unique k‐mers in the reference database increases.

### 3.7 Performance of Analysing True Ancient DNA Metagenomes

Between 0.8% and 5.6% of the reads from the three sedaDNA datasets were annotated by either workflow (Figure 5). On average, *wholeskim*‐unassembled annotated 2.48× more reads than *Holi*‐assembled for all three datasets. Each taxon present in the final annotations had more reads identified by *wholeskim*‐unassembled than by *Holi*‐assembled. The list of identified taxa is largely consistent between the two workflows with all taxa identified by *Holi‐*assembled also being identified by *wholeskim*‐unassembled. However, there are seven instances of a taxon being identified as present by *wholeskim*‐unassembled, while having too few reads (*r* < 0.01% of query reads) to be retained by *Holi*‐assembled in the final annotations. From the Greenland permafrost sample, 870,863 reads were taxonomically annotated to the *Betula* genus and 799,486 of these reads were mapped to the *B. pendula* genome assembly. The mode length of these mapped reads was 55 bp and the percent of damage substitions at the read termini was 31.2% dropping sharply from the read termini (Figure S5).

## 4 Discussion

The *wholeskim*‐unassembled workflow is currently the only approach to accurately annotating DNA metagenomes that allows for the effective indexing and querying of large‐scale unassembled genome skim datasets. This workflow correctly annotated 1.16× more simulated reads and generally annotated 2.48× more *seda*DNA reads than the existing metagenomic DNA annotation workflow used on large‐scale genome skim datasets, *Holi* (Pedersen et al. 2016; Wang et al. 2021). The *wholeskim*‐unassembled workflow is, however, slightly more prone to erroneous annotations, with 1.58× more assignments to taxa outside the genus of interest; however, these misassignments only totalled 0.27% of the total simulated read dataset and are distributed between many taxa. This misassignment rate is close to the reported rate of 0.24% from the simulated data in *Holi*'s original publication (Pedersen et al. 2016).

The *Holi*‐assembled and *wholeskim*‐unassembled workflows operate differently and only jointly identify 27.5% of the total correctly assigned simulated reads. The discrepancy in assignment performance and subset of reads annotated between the workflows could be attributed to two different factors: the method of reference database construction or the matching algorithm itself. When *wholeskim* was run using the assembled contigs database used with the *Holi* pipeline, it misassigned considerably more reads (Figure S4), as expected, as each pipeline is tailored to its corresponding reference database composition.

One possible source of this misassignment rate could be the off‐target sequencing of the reference herbarium samples (Figure 2). If the off‐target read originates from the target taxon's natural metagenomic community, the taxonomic annotation remains correct. However, it has been demonstrated that herbium samples develop a postmortem microbial community that could complicate this process (Bieker et al. 2020). We hypothesize that these microbial reads, if not filtered out by *wholeskim* as contamination, would be shared among multiple taxa resulting in an LCA and annotation at a high taxonomic level which would not contribute to the misassignment rate. Aquatic taxa are shown to have higher levels of contaminate reads which could originate from their surroundings when sampled and may result in higher false positive rates for *seda*DNA samples collected in this environment.

There was a mode 10× loss of information when assembling the genome skims into contigs (Figure 3A). With the Illumina HiSeq 2500 having a sequencing error rate of 1.12e^−3^, *σ* = 5.44e^−3^ (Stoler and Nekrutenko 2021), some of these discarded k‐mers are a product of sequencing error; however, many are also likely from low‐coverage regions that were unable to be assembled. Query reads spanning these low‐coverage areas would not be able to be identified by the *Holi*‐assembled workflow, but could be annotated by *wholeskim*‐unassembled. Conversely, query reads with a single sequencing error near the centre of the read would have a very low number of shared k‐mers with the reference index in the *wholeskim‐*unassembled workflow. These reads would, however, be able to be identified by bowtie2's mapping‐based approach in *Holi*‐assembled.

**FIGURE 6:**
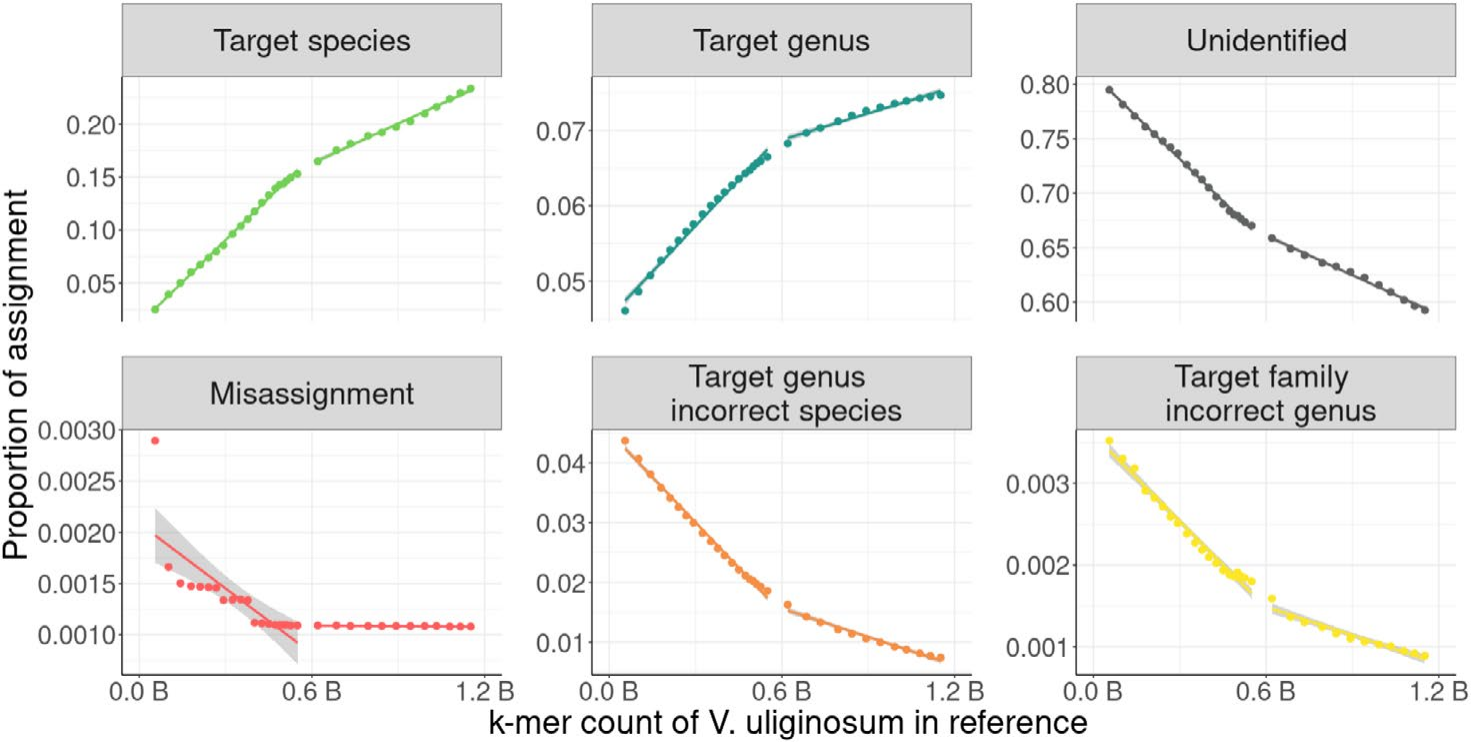
The taxonomic assignment of 200 k simulated *Vaccinium uliginosum* reads as increasing amounts of unique k‐mers of *V. uliginosum* are added to the reference database. Note the varying y‐axis scales. Lines of best fit are added to points before and after estimated 1× coverage of the *V. uliginosum* genome (~0.6 B k‐mers).

As a genome skim's depth of coverage increases past 1×, the number of unique k‐mers continues to increase, albeit at a smaller slope (Figure 3B). These are likely “erroneous” k‐mers being included in the reference dataset, but their inclusion does allow for more accurate assignment of reads (Figure 6). The mechanism behind this improvement could be that the spurious k‐mers allow for some “fuzzy” matching to compensate for sequencing errors in the query reads as well as individual genomic variation. Other k‐mer based metagenomic tools that have incorporated ‘fuzzy’‐matchingreported higher accuracy assignments when compared to exact‐matching k‐mer tools (Firtina et al. 2023). It does not appear that incorporating these spurious k‐mers in the reference database has significantly increased incorrect assignment to these taxa since *V. uliginosum* has the highest genome coverage and unique k‐mer count, but is not detected as one of the false positives by *wholeskim*‐unassembled in the simulated read datasets. The slightly higher misassignment rate of *wholeskim*‐unassembled could be attributed to the hash collisions inherent in the probabilistic Bloom filter data structure (Bloom 1970). The false positives produced by this mechanism are expected to be distributed among all taxa in the database at a low frequency so they are largely filtered out by employing a cutoff of the minimum proportion of reads assigned to a taxon if that taxon is to be retained in the final dataset (*r* = 10^−5^). Through *in silico* testing with simulated datasets, *Holi* arrived at a similar but less stringent threshold (*r* = 10^−5^ for all assigned reads, rather than queried reads) for discarding false positive taxa attributed to sequencing errors, amplification errors, or DNA damage (Pedersen et al. 2016). With both workflows, misassignments generally cluster around closely related taxonomic groups with the majority being congeneric species and confamilial taxa (Figure S6). These misassignments could be the result of conserved genomic regions that have not been sequenced in one of the low‐coverage genome skims. An example can be seen in GB5, one of the ancient genomic samples, where a small proportion of the reads were assigned to the cultivated plant *Hordeum* although they probably represent the local native species *Leymus* (Koamtsu et al. 2024). *Hordeum* was assigned a proportion of reads marginally above the cutoff (0.012%), while the family containing *Hordeum*, *Hordeinae*, was assigned a proportion of reads an order of magnitude larger (0.85%). This highlights the shortcomings of using a fixed cut‐off (r) for retaining taxa when the DNA content of a metagenome is not expected to be equally distributed among all taxa present in a sample. Instead, the cutoff could be a proportion of the most dominant taxon and take into consideration taxonomic distance.

A major challenge for metagenomic annotation is the taxonomic completeness of reference databases (L. Parducci et al. 2019; Wang et al. 2021). When queried with simulated reads from species absent in the reference database, both workflows produced few misassignments, suggesting that while reference database incompleteness produces false negatives, it is not a major contributor to false positives. While some taxa have a majority of reads assigned to species level in the *wholeskim*‐unassembled workflow (e.g., *V. uliginosum* ), other taxa like *Betula nana* have a majority assigned to the genus‐level, with significant portions assigned to congeneric species, making a species level identification unreliable, especially in an environmental sample with many taxa present (Figure S7). The authors of the *Holi* pipeline have also recognised this limitation and generally identify flora to the genus‐ or family‐level, except when combined with species distribution information (Pedersen et al. 2016; Wang et al. 2021). This taxonomic resolution is one of the major limitations of shotgun‐sequenced metagenome annotation when compared to other methods like metabarcoding (Revéret et al. 2023).

**FIGURE 7:**
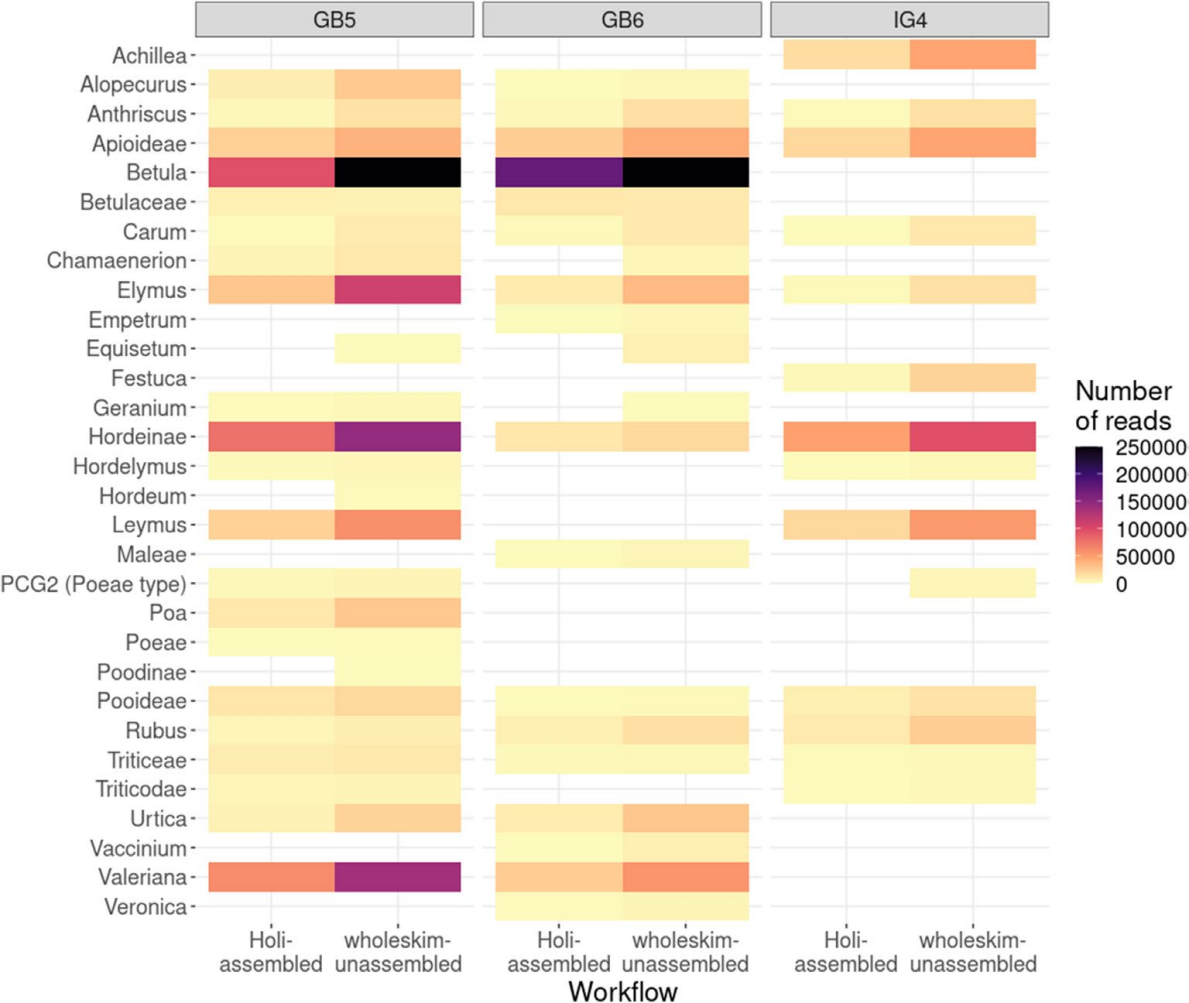
The number of reads annotated by the *Holi‐*assembled and *wholeskim*‐unassembled workflows of three shotgun‐sequenced *seda*DNA samples.

Earlier *seda*DNA metagenomic studies using primarily the NCBI nt or RefSeq databases as references reported very low proportions of reads identified to any taxonomic level of *Viridiplantae*, with (Slon et al. 2017) identifying a mean of 0.07%, (Courtin et al. 2022) identifying 0.05%, and (Parducci et al. 2019) identifying only 0.0002% of queried reads. The first application of the *Holi* pipeline using NCBI nt reports 0.05% of total reads assigned to some level of *Viridiplantae* (Pedersen et al. 2016). The addition of the PhyloNorway genome skim contigs to the *Holi* pipeline gave a significant increase to the number of reads annotated to *Viridiplantae*, 1.7% (Wang et al. 2021). This percentage of identified reads is consistent with how many reads were annotated by *Holi*‐assembled for the true *seda*DNA datasets in this paper, 0.8%–2.3%, while *wholeskim*‐unassembled assigned 2.1%–5.6% for the same datasets. Other comparisons between metagenomic annotation tools, including *Holi*, show that they largely agree on the taxa present in datasets (Harbert 2018). Here, we report the same as 51/58 taxa observations in the *seda*DNA datasets are shared between workflows (Figure 7). While *wholeskim*‐unassembled is not able to identify reads with a mismatch near the centre of the fragment, errors near the ends of the fragment do not adversely affect the k‐mer similarity score as strongly. Since ancient DNA deaminations typically occur at the ends of fragments (Dabney et al. 2013), *wholeskim*‐unassembled still annotated a large proportion of the *seda*DNA reads exhibiting ancient DNA damage as demonstrated on *Betula* reads annotated from a two million year old permafrost sample (Figure S5).

## 5 Conclusion

By incorporating information from the entire low‐coverage genome skims, the *wholeskim*‐unassembled workflow is able to accurately annotate more reads than other metagenomic pipelines. It is clear that increasing the taxonomic coverage of the reference database reduces the number of false negatives, but we also demonstrate that it does not greatly impact the number of false positive annotations in *wholeskim*‐unassembled. Similarly, increasing the genomic coverage of the genome skims used as reference increases the number of annotated reads, but with diminishing returns after ~1× depth of coverage. Since *wholeskim*‐unassembled and *Holi*‐assembled are correctly annotating different sets of reads, their combined use results in the largest number of reads for applications such as metagenomic assembly. However, if the intent of the study is to infer the community composition of the sample, using only the more computationally efficient *wholeskim*‐unassembled is sufficient.

## Author Contributions

L.E., F.B., I.G.A. and E.C. conceptualised the study. T.L. designed and coded the software kmindex. L.E. wrote the code for wholeskim with feedback from F.B. and E.C. Collection and genome skimming of the nine additional species was performed by L.E. Benchmarking of the pipeline was performed by L.E. Interpretation of data was done by L.E. with input from I.G.A. and E.C. The manuscript was written by L.E. with feedback from all co‐authors.

## Conflicts of Interest

The authors declare no conflicts of interest.

## Supporting information


Figures S1‐S7.

## Data Availability

The open‐source *wholeskim* pipeline is available at https://github.com/ArcEcoGen/wholeskim. Simulated datasets are accessible at DRYAD while the aDNA dataset is available at ENA accession number.
