# Supplementary material for: wholeskim: Utilising Genome Skims for Taxonomically Annotating Ancient DNA Metagenomes
Source: Mol Ecol Resour. 2025 Jul 21;25(8):e70001. doi: 10.1111/1755-0998.70001 (PMC12550482; doi:10.1111/1755-0998.70001)
Supplement: Supplementary file 1 — Figures S1‐S7. [file MEN-25-e70001-s001.docx]

# Supplementary information


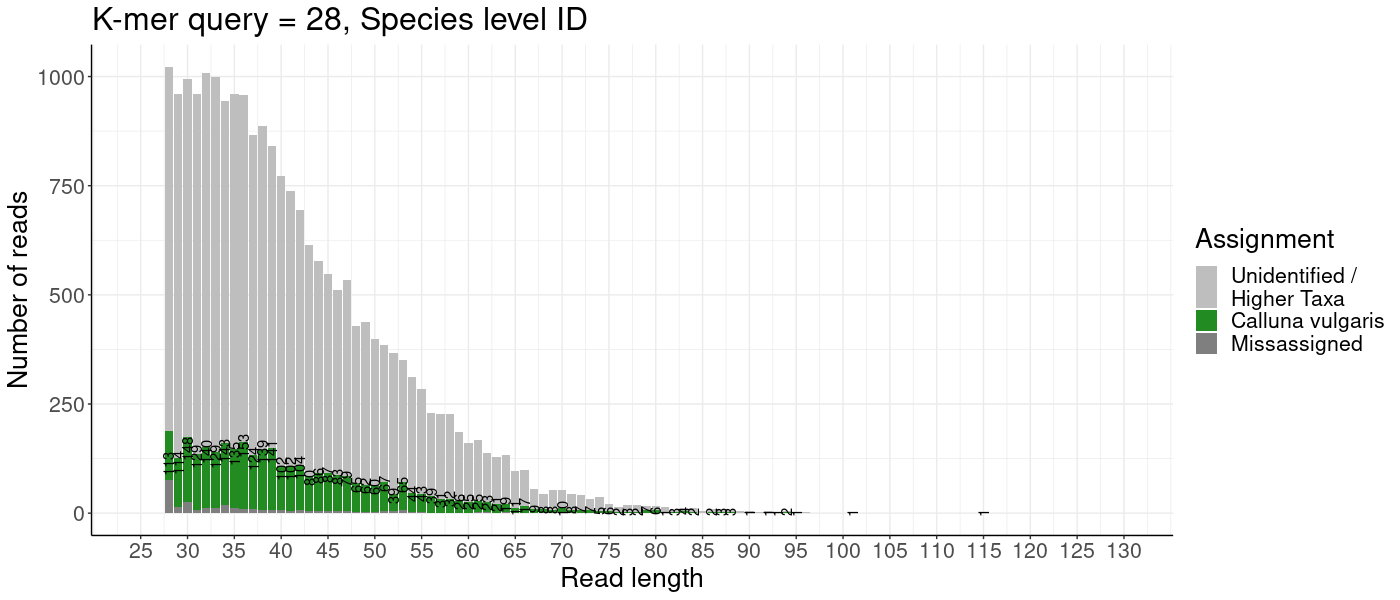

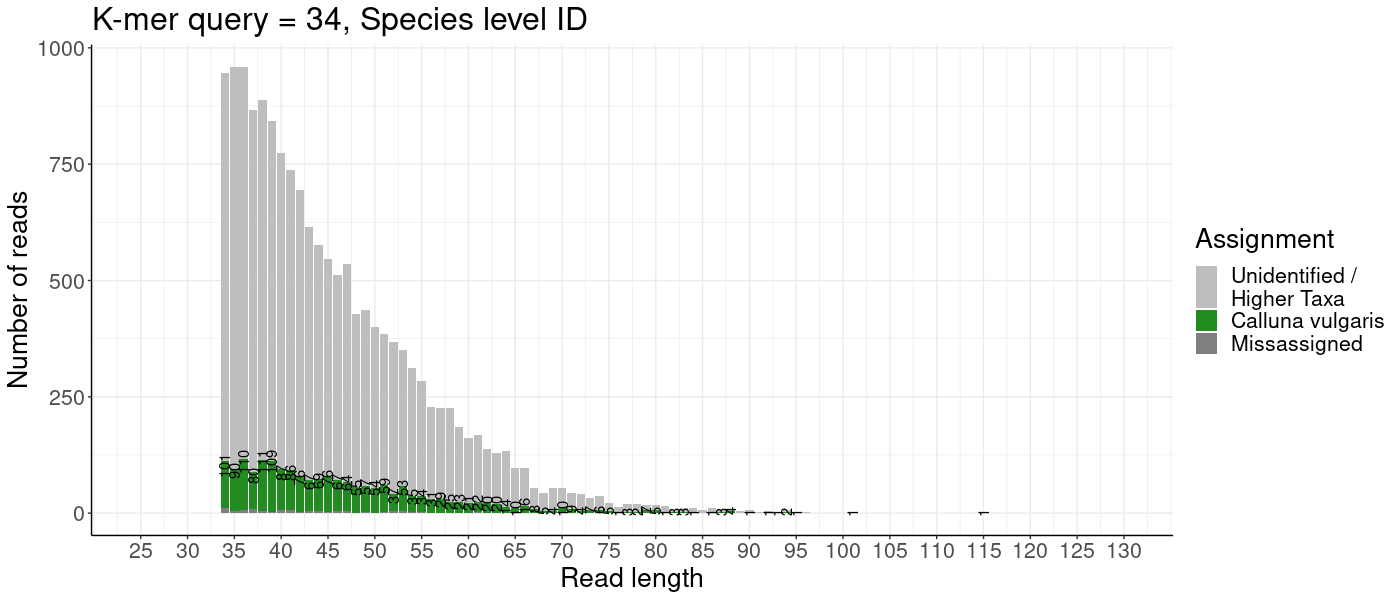


Supplementary Figure 1. The assignment accuracy of k-mer = 28 and k-mer = 34 of simulated *Calluna vulgaris* reads by the *wholeskim*-unassembled workflow. Using an effective k-mer size of 28 is able to assign more reads, but has a significantly larger misassignment rate than a k-mer size of 34.


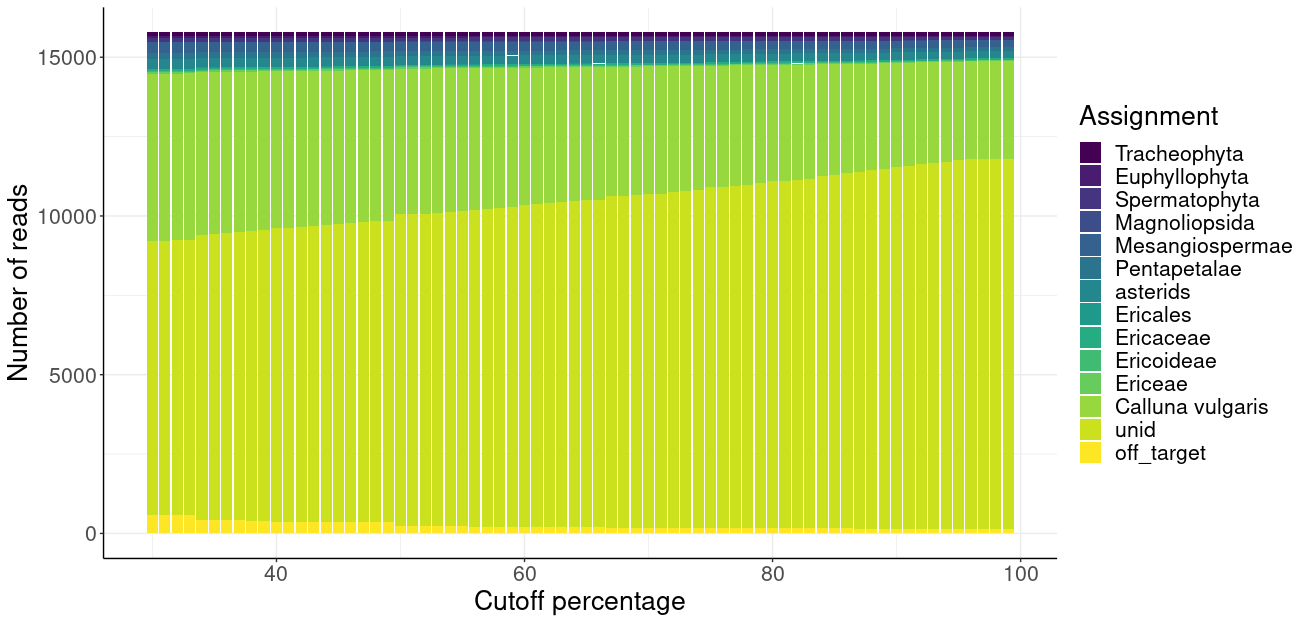
Supplementary Figure 2. The assignment of simulated *Calluna vulgaris* reads by the *wholeskim*-unassembled workflow with varying cutoff levels (t). A cutoff value of 70% provides the most favorable ratio of *Calluna vulgaris* assigned reads to off-target assigned reads (22.9). Note that the taxonomic assignment cutoff (r = 0.0001) is not applied here to the misassigned reads.


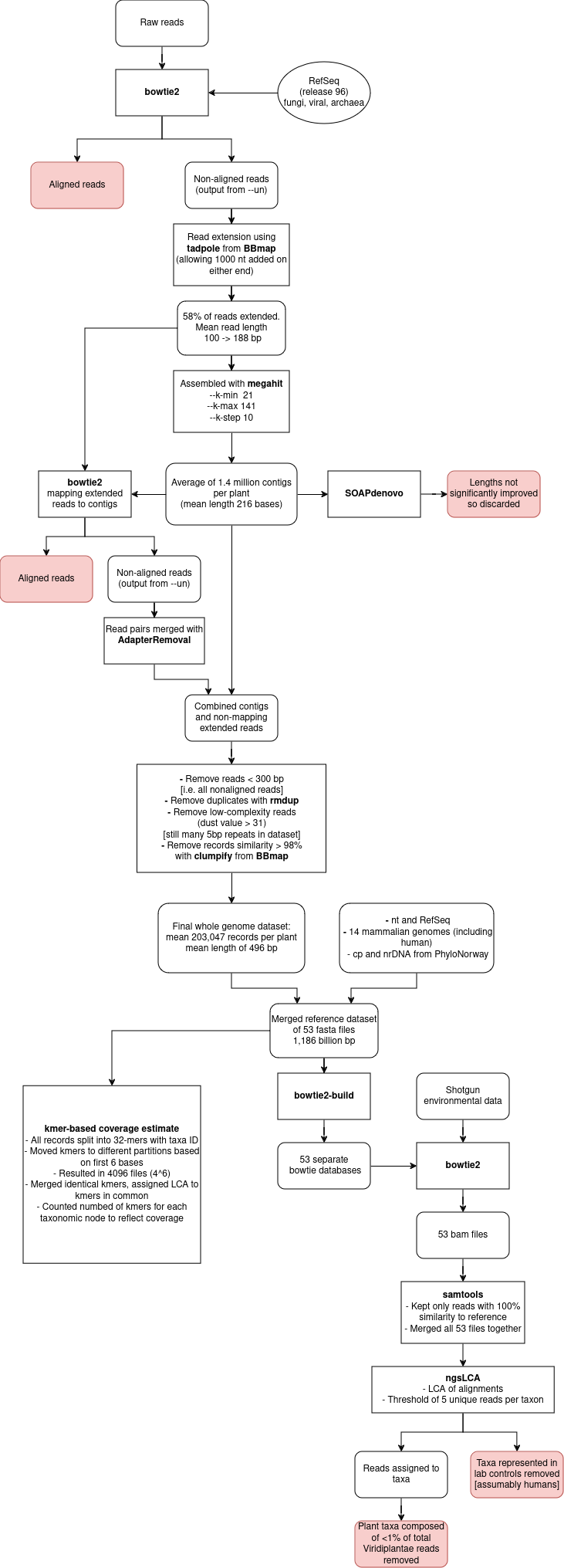


Supplementary Figure 3. A diagrammed workflow of the PhyloNorway genome skims processed by the *Holi* pipeline as applied in Wang et al. 2021.


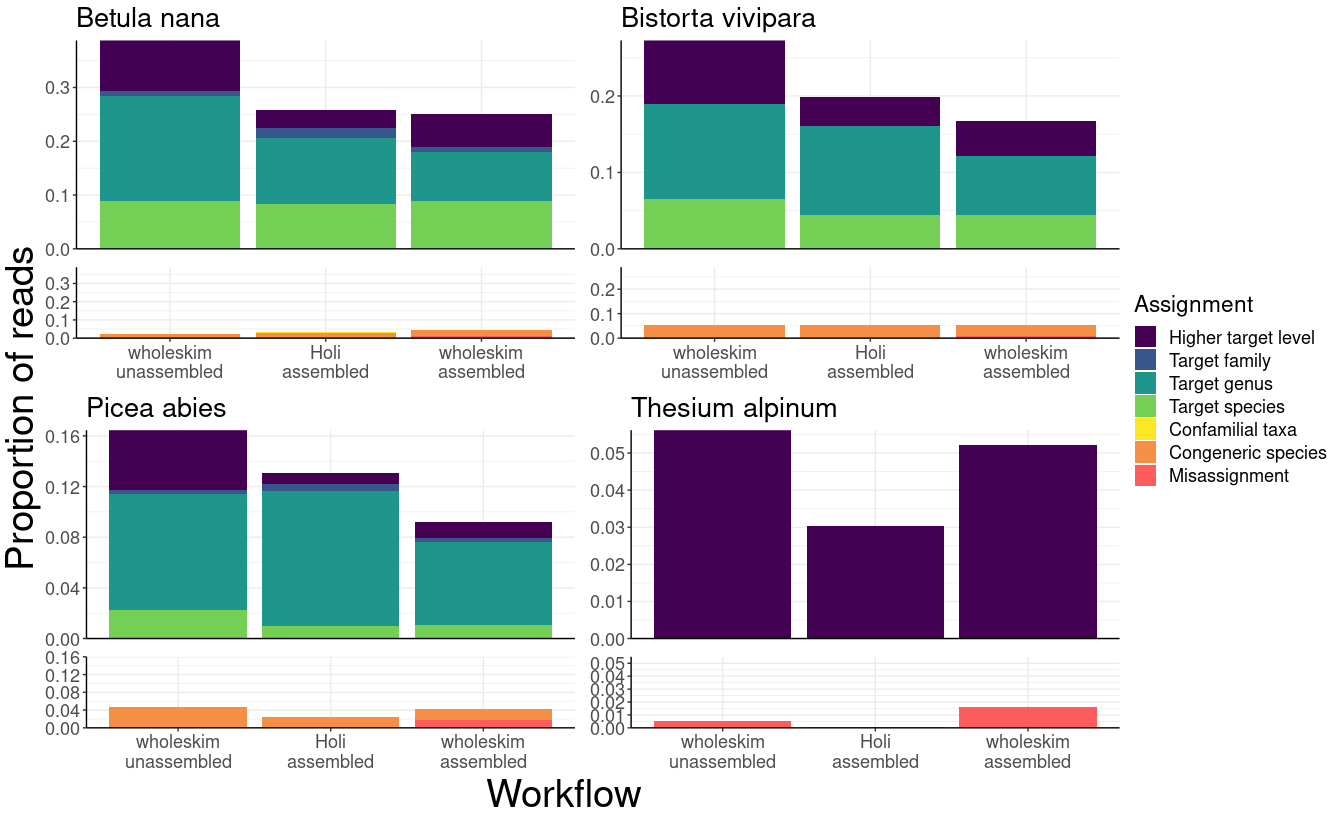


Supplementary Figure 4. Assignment accuracy of simulated reads including the *wholeskim*-assembled workflow. Note the variable y-axis scales between species.


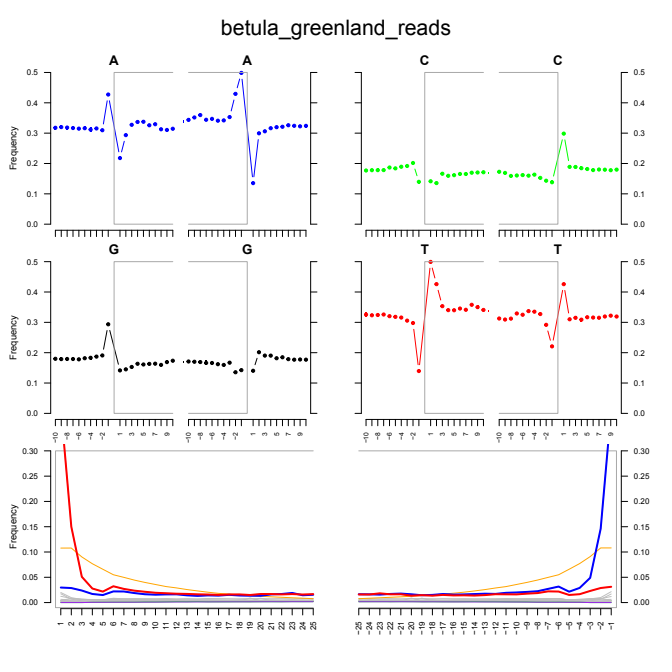
 Supplementary Figure 5. The fragment misincorporation plot of the *Betula* annotated reads from sample 119_B3_116_L0 mapped to the assembled *B. pendula* genome produced through mapDamage (Jonsson et al. 2013).


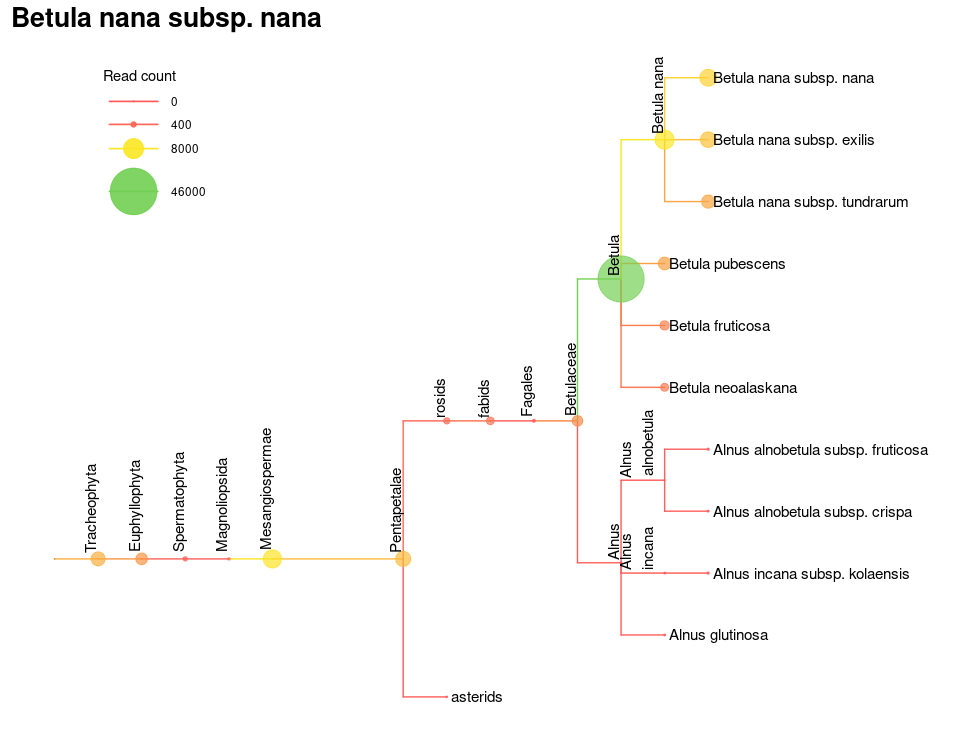


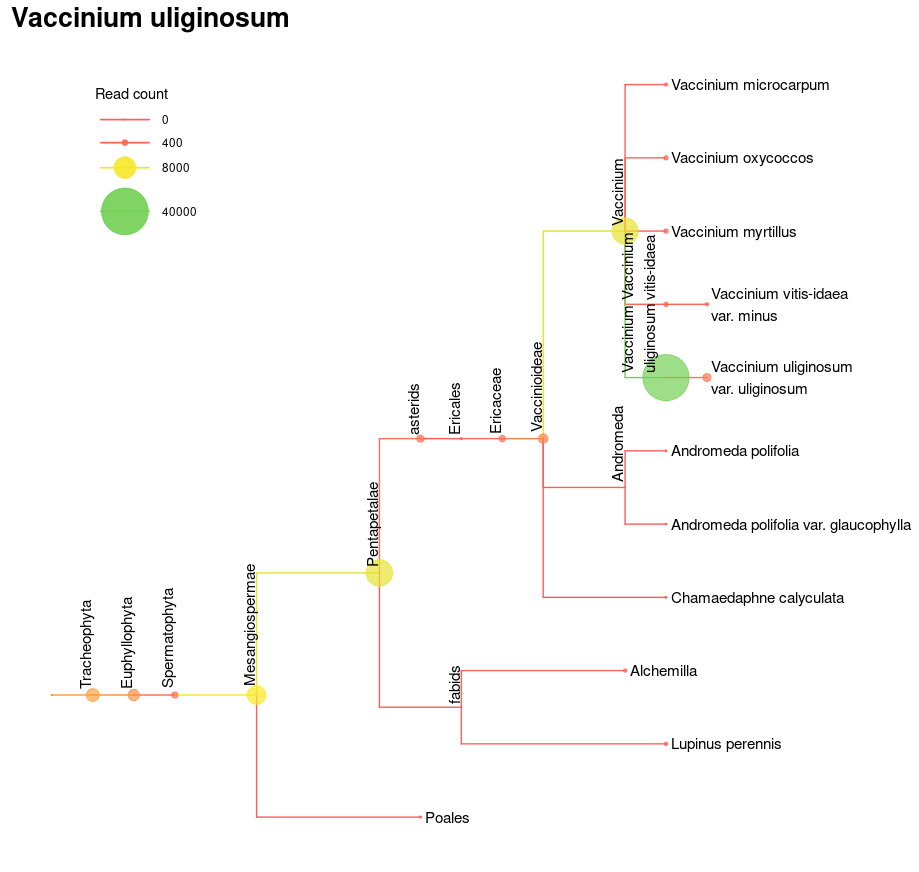


Supplementary Figure 6. Phylogenetic tree of assigned simulated reads for *Betula nana* (A) and *Vaccinium uliginosum* (B). Both the color and size of the node are proportional to the number of reads assigned to that taxon.


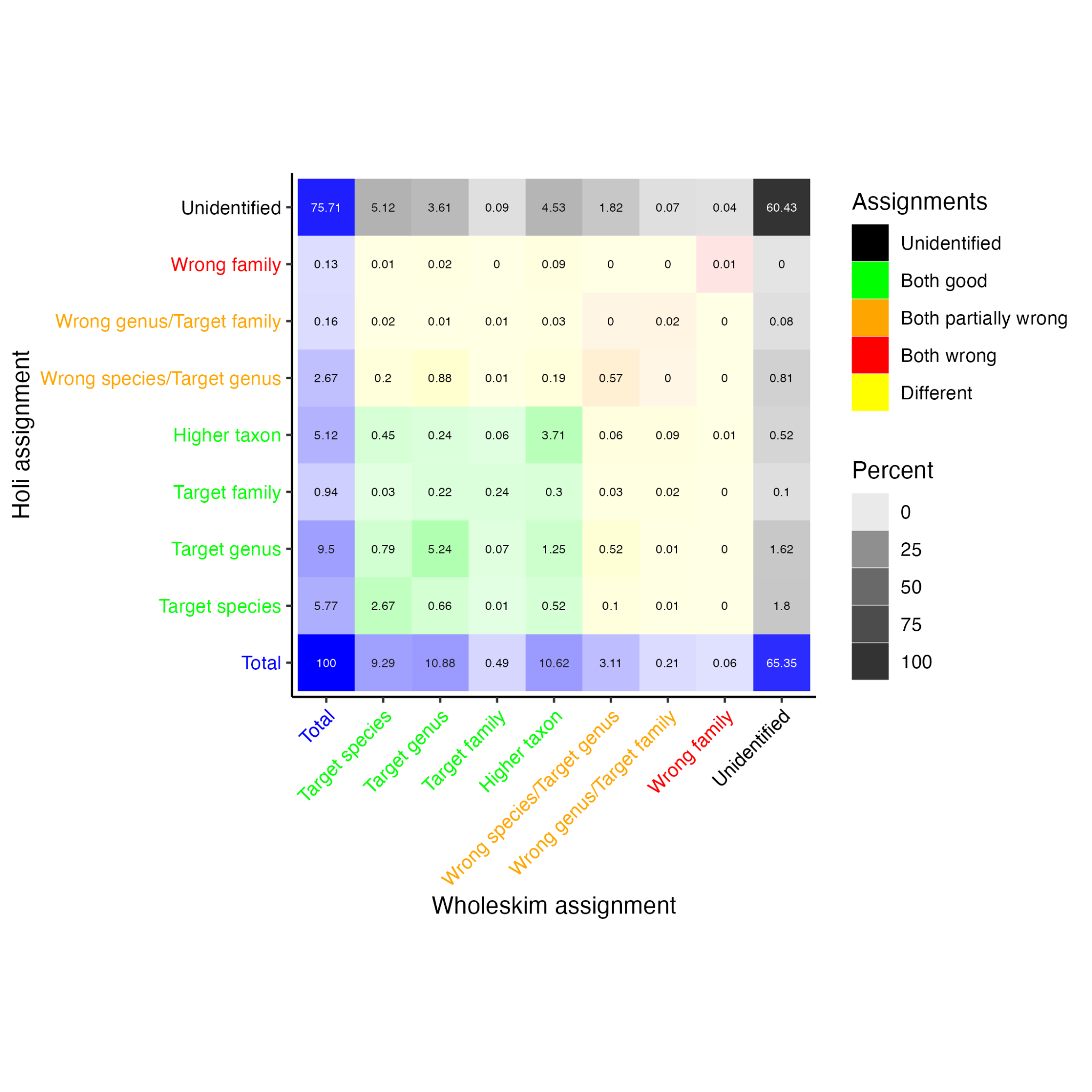


Supplementary Figure 7: A confusion matrix of the cumulative assignments for all nine test species present in PhyloNorway. The confusion matrix presented here illustrates how the same read can have different accuracy of assignment from one pipeline to another.

Identifications made on the taxonomic assignment by both the pipelines can be categorized according to their quality. The total columns and row present the intrinsic results respectively of Wholeskim and Holi. All values are expressed in percent of the annotated reads.
